# Supplementary material for: Network percolation reveals adaptive bridges of the mobility network response to COVID-19
Source: PLoS One. 2021 Nov 9;16(11):e0258868. doi: 10.1371/journal.pone.0258868 (PMC8577732; doi:10.1371/journal.pone.0258868)
Supplement: S1 File — (PDF) [file pone.0258868.s001.pdf]

Supporting Information for

Network percolation reveals adaptive bridges of the mobility network response to COVID-19

S1 Daily Device Number Variation

Fig. S1 shows the total daily active devices in the network, and we can observe a steady decrease from over 17 million devices per day to 15 million during the study period. The change suggests a sample reduction with the COVID-19 outbreak, yet the quantity change is insignificant compared to mobility reduction.

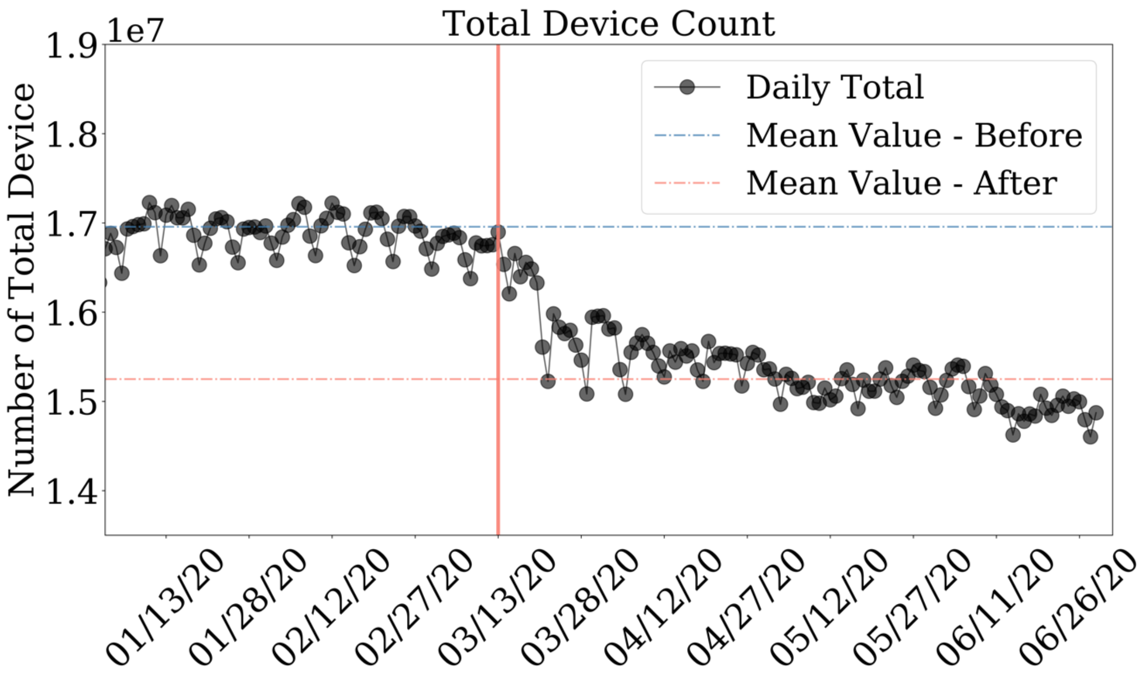

Fig. S1. Daily Total Device Count Aggregated across all Counties

S2 Data Representativeness Validation

In Fig. S2, we show the correlation between the number of users identified in each county and the population from 2018 ACS data is high (Pearson $r > 0.94$) throughout the study period on the county level. Despite a minor decrease in the sample size, we validate that the data is instrumental in describing human mobility at the county level and studying the COVID-19 pandemic.

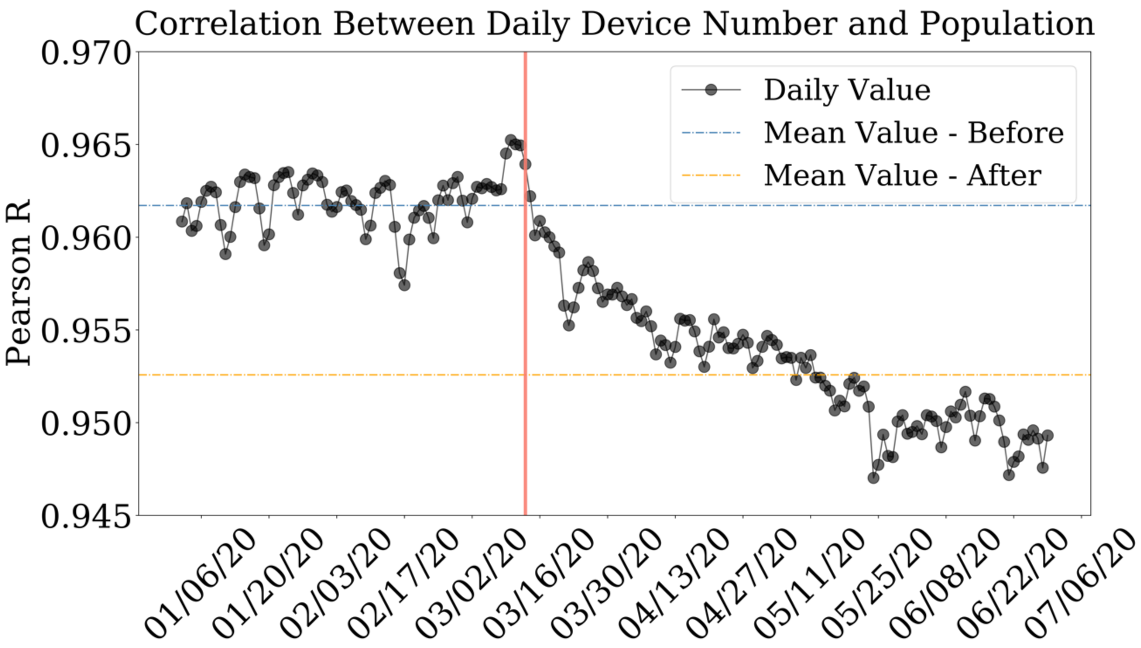

Fig. S2. The Daily Correlation Between County Population and Identified Device Number

S3 Daily Fluctuation of Percolation Criticality

Since the human travel behavior can be affected by day-of-the-week effects, we construct the aggregated network by averaging networks from consecutive seven days in this study to mitigate such issue. For any two nodes (i.e., counties) *i* and *j*, the average link weight *w* on day *k* is $(\sum_{-3}^{3} w_{i,j,k+n})/7$. We then build both directed and undirected graphs using the 7-day average flux of each date to address the weekday/weekend effect. and we show the difference of the network structure at percolation criticality of both directed and undirected graphs on randomly selected 12 different days below. Here we can see that some daily variations in mobility flux could lead to the separation of certain sub-components of the giant components. We show the difference of percolation critical threshold and components between undirected (Fig. S3, S4) and directed networks (Fig. S5, S6). We can observe the resemblance of a silhouette of the large components and the neighboring counties, which suggests the similarity between both scenarios. Larger components could be seen in Fig. S4 which is due to that the critical threshold for the directed being much smaller than undirected.

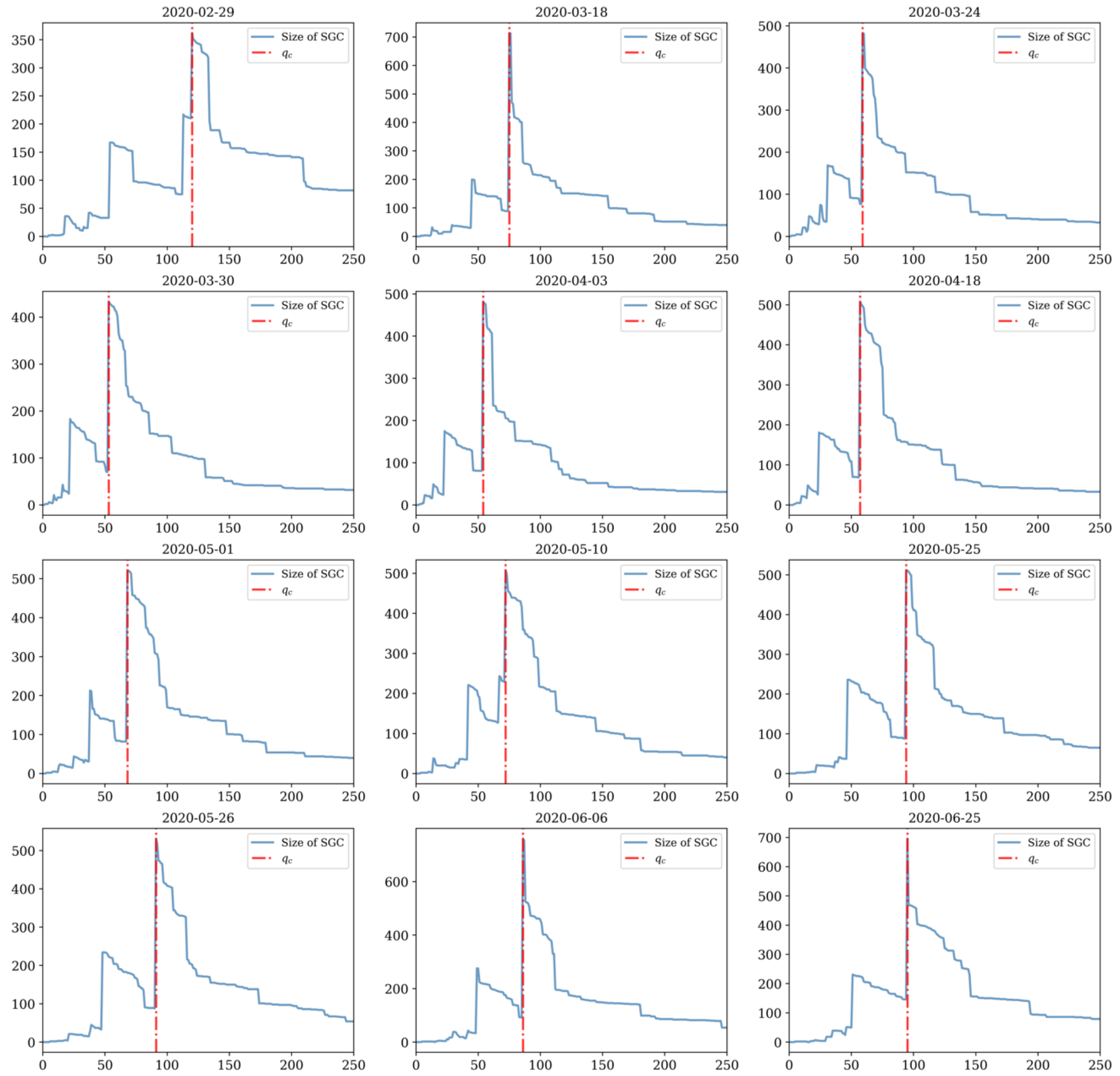

Fig. S3. The Variation of Critical Threshold of Undirected Network across Different Days

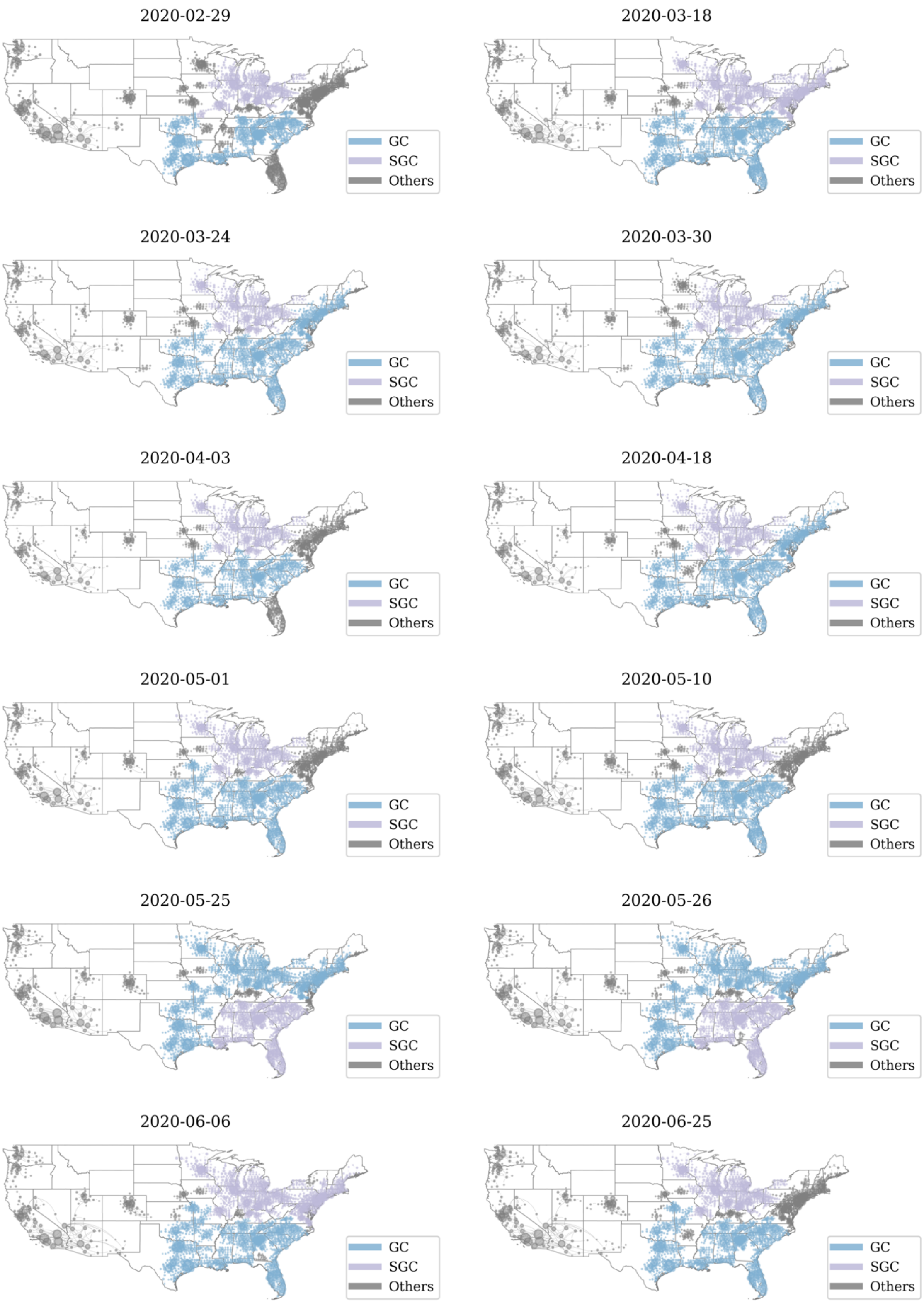

Fig. S4. The Variation of Detected Component Structure at Critical Threshold of Undirected Network across Different Days

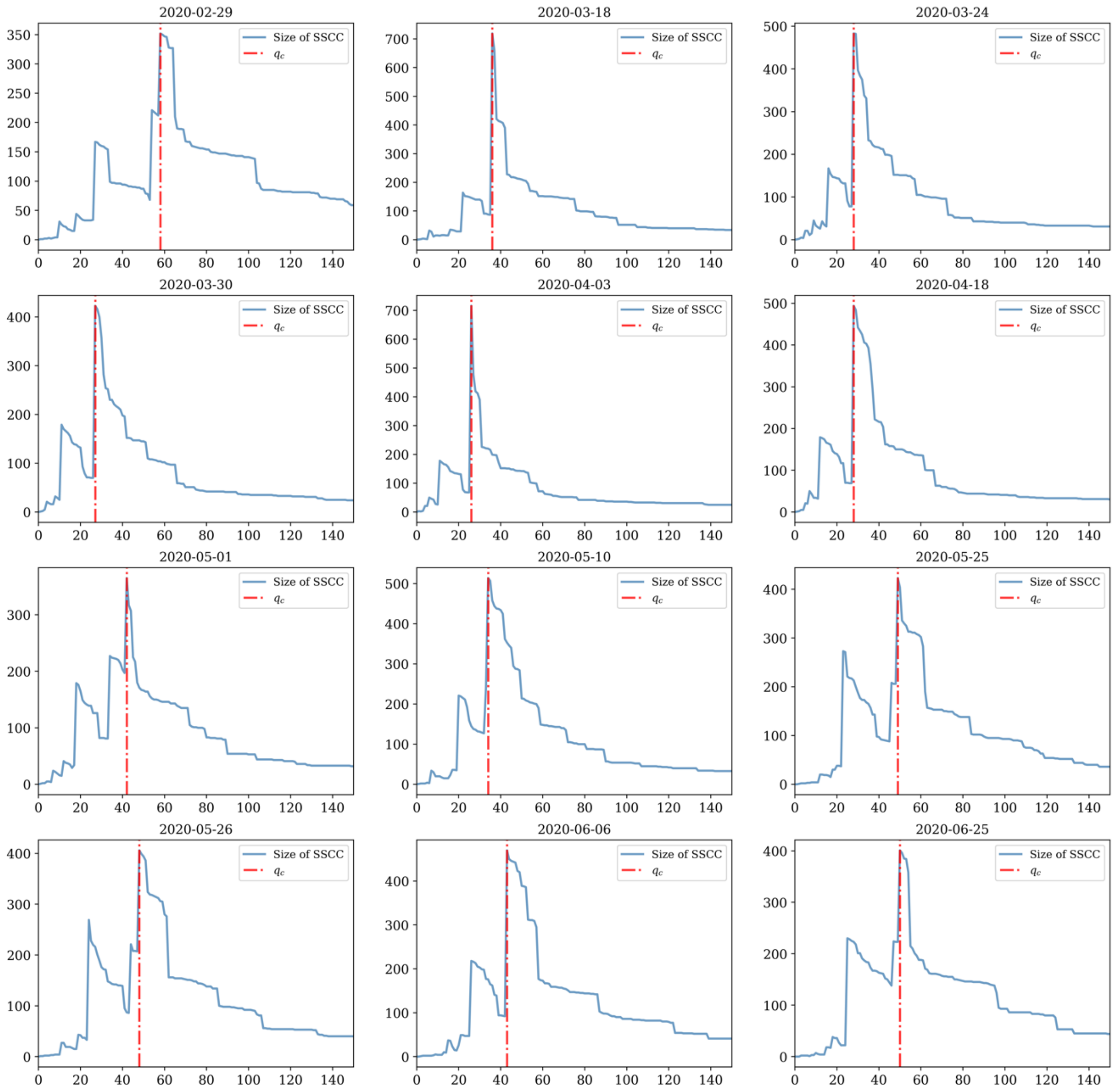

Fig. S5. The Variation of Critical Threshold of Directed Network across Different Days

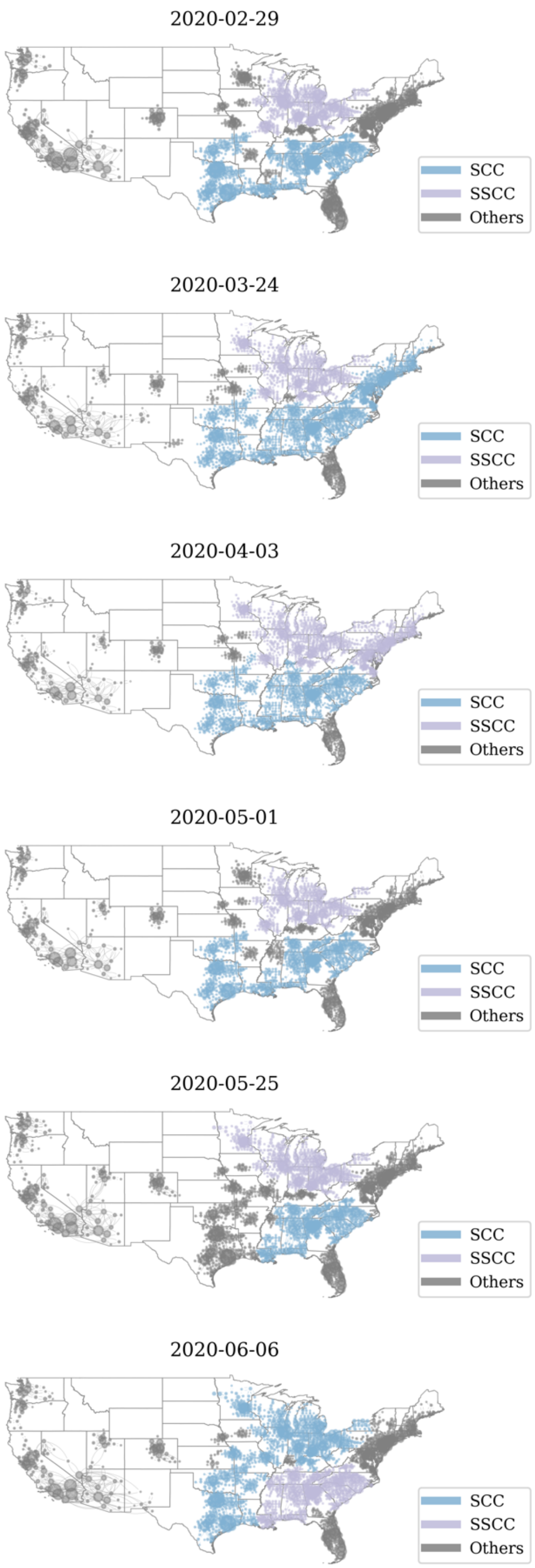

Fig. S6. The Variation of Detected Component Structure at Critical Threshold of Directed Network across Different Days

S4 Critical Link Determination

In order to quantify the significance of the recurrence of the critical links, we compute the number of days each critical link appears at the threshold of criticality divided by the total number of unique days across the study period (190 days). The x-axis in Fig. S7 is the threshold of the recurrence rate, while the y-axis shows the overall numbers of critical links correspond to the threshold. We can see that as the threshold increases, the number of links drops accordingly for both GC and Non-GC links. However, for the Non-GC links, the number drops significantly with the threshold between 0 and 0.1, indicating a level of randomness in the critical links. At the threshold of 0.1, the overall link number becomes stable. We see the randomness for the GC links is much less severe, and the decrease is stable without any significant plummet. Therefore, we use 0.1 as the threshold for the eligibility criterion: for a link to be considered critical, it needs to appear at least 10% of the total number of different days during each stage of the COVID-19 pandemic (see Fig. 4 in the main text).

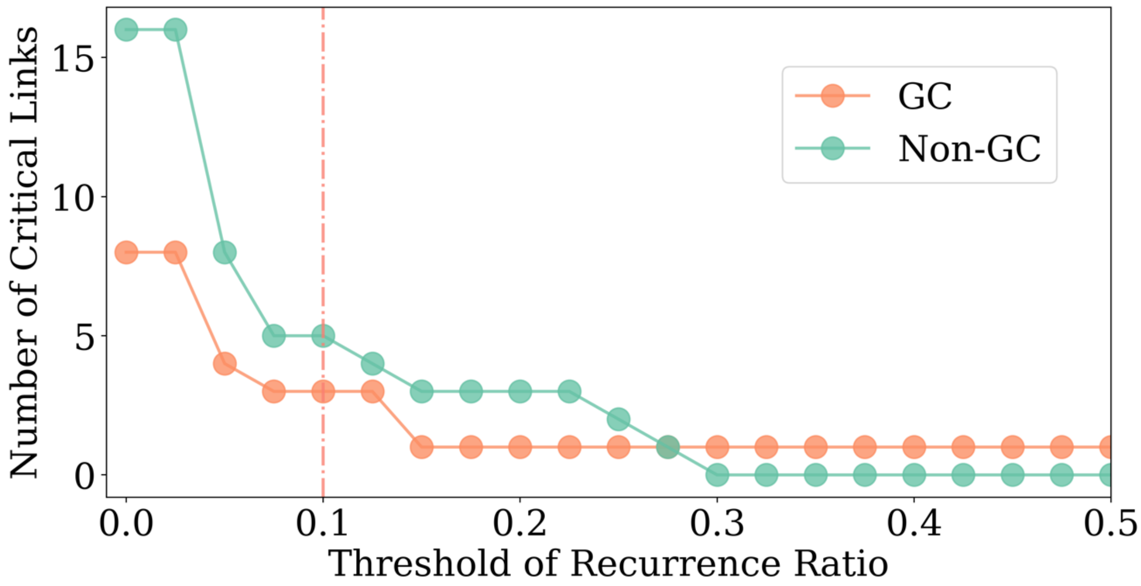

Fig. S7. The Threshold of the Recurrence Rate versus the Number of Critical Links

The Evolving Pattern of Critical Links

Node 1	Node 2	Overall	Stage-0	Stage-1	Stage-2	Stage-3
Pulaski - MO	Laclede - MO	0.52	0.65	0.47	0.09	0.53
Nolan - TX	Mitchell - TX	0.26	0.00	0.24	0.00	0.44

Doña Ana - NM	Maricopa - AZ	0.25	0.15	0.41	0.68	0.38
Huntingdon - PA	Juniata - PA	0.22	0.65	0.00	0.18	0.00
Bradford - PA	Chemung - NY	0.15	0.00	0.06	0.77	0.15
Webster - MO	Laclede - MO	0.13	0.25	0.47	0.00	0.00
St. Martin - LA	Iberville - LA	0.13	0.00	0.06	0.00	0.21
Nelson - VA	Amherst - VA	0.13	0.00	0.24	0.00	0.21
Luna- NM	Maricopa - AZ	0.09	0.20	0.06	0.18	0.09
Seneca - NY	Cayuga - NY	0.05	0.05	0.00	0.09	0.06
Steuben - NY	Livingston - NY	0.05	0.00	0.00	0.00	0.12
Bradford - PA	Tioga - NY	0.05	0.00	0.06	0.00	0.03
Luna - NM	Riverside - CA	0.03	0.00	0.00	0.18	0.00
Tioga - NY	Chemung - NY	0.02	0.15	0.00	0.00	0.00
Doña Ana - NM	Bernalillo - NM	0.02	0.10	0.00	0.00	0.00
Erath - TX	Eastland - TX	0.02	0.15	0.00	0.00	0.00
Taylor - TX	Coleman - TX	0.01	0.10	0.00	0.00	0.00
Franklin - MO	Camden - MO	0.01	0.00	0.00	0.00	0.06

Table S1. The Detailed Node Information Ranked by Frequency of the Undirected Critical Links

Node 1	Node 2	Overall	Stage-0	Stage-1	Stage-2	Stage-3
Laclede - MO	Pulaski - MO	0.23	0.24	0.07	0.23	0.14
Nolan - TX	Mitchell - TX	0.23	0.00	0.00	0.00	0.29
Pulaski - MO	Laclede - MO	0.22	0.35	0.47	0.09	0.09
Luna - NM	Maricopa - AZ	0.17	0.00	0.20	0.86	0.09
Maricopa - AZ	Doña Ana - NM	0.16	0.06	0.33	0.36	0.26
Maricopa - AZ	Luna - NM	0.15	0.06	0.33	0.59	0.12

Huntingdon - PA	Juniata - PA	0.15	0.47	0.07	0.00	0.00
Bradford - PA	Chemung - NY	0.14	0.00	0.13	0.45	0.21
Mitchell - TX	Nolan - TX	0.13	0.00	0.00	0.00	0.26
Amherst - VA	Nelson - VA	0.10	0.00	0.00	0.05	0.26
Webster - MO	Laclede - MO	0.09	0.35	0.00	0.00	0.00
St. Martin - LA	Iberville - LA	0.09	0.00	0.06	0.00	0.21
Pinal - AZ	El Paso - TX	0.07	0.00	0.27	0.27	0.00
Cayuga - NY	Seneca - NY	0.06	0.12	0.00	0.00	0.09
Chemung - NY	Bradford - PA	0.05	0.00	0.07	0.23	0.03
Nelson - VA	Amherst - VA	0.04	0.00	0.07	0.00	0.15
Seneca - NY	Cayuga - NY	0.04	0.06	0.00	0.05	0.06
Livingston - NY	Steuben - NY	0.04	0.00	0.07	0.00	0.09
Laclede - MO	Webster - MO	0.04	0.00	0.20	0.00	0.00
Doña Ana - NM	Maricopa - AZ	0.03	0.06	0.00	0.05	0.09
Chemung - NY	Tioga - NY	0.03	0.12	0.00	0.00	0.00
Erath - TX	Eastland - TX	0.02	0.18	0.00	0.00	0.00
Chaves - NM	Lincoln - NM	0.02	0.06	0.00	0.00	0.06

Table S2. The Detailed Node Information Ranked by Frequency of the Directed Critical Links

S5 The Critical Links are not random

We use the days before the national emergency state for demonstration. A 7-day sliding window has been applied to all days with an average flux as edge weight, and then we detect the critical link. We then implement random shuffling, where we randomize the network 100,00 times and find the bottleneck links with the same method at each instance. Fig. S8 compares the overall recurrence rate of the original network with three randomized networks (see Fig. 5 in the main text). The recurrence rate is an indicator of the overall significance of the links based on their likelihood to emerge as critical bridges on multiple days. We can see that based on the Zipf plot of the recurrence rates of links, the top-ranked links appear with an occurrence rate greater than 0.4 for the original networks. Such high reoccurrence demonstrates the robust percolation feature of the travel network. In contrast, all the links at different randomization scenarios appeared with a remarkably low probability of recurrence (less than 0.01). The difference suggests that random networks are difficult to control with unstable bridges. Meanwhile, this also highlights the non-random yet dynamical nature of the original network's bottle links since their appearances are related to the network topology determined by the spatial and demographical information as well

as the mobility response to the COVID-19.

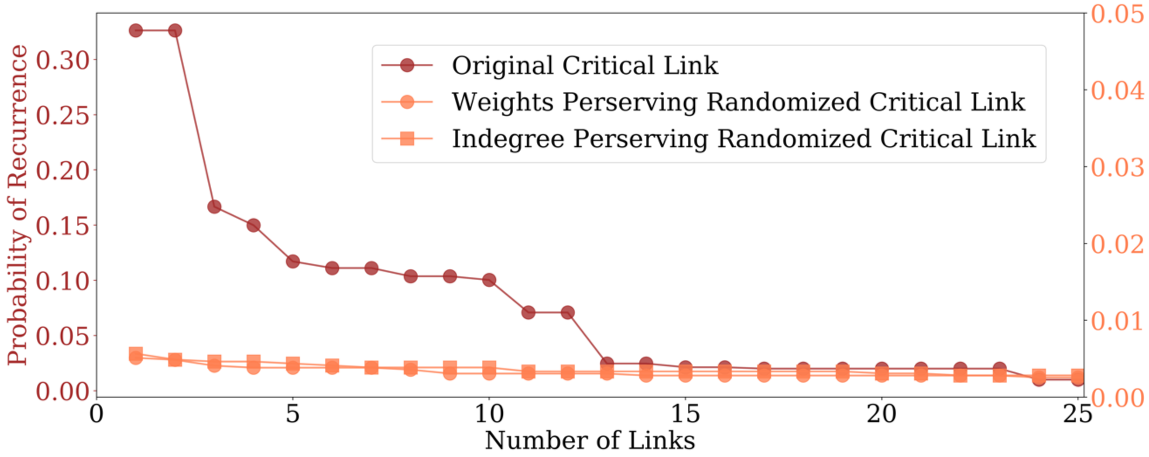

Fig. S8. The Comparison of the Recurrence Rate between the Weight Randomized Network and Original Network

SI References

1. D. Li, B. Fu, Y. Wang, G. Lu, Y. Berezin, H. E. Stanley, S. Havlin, Percolation transition in dynamical traffic network with evolving critical bottlenecks, Proceedings of the National Academy of Sciences 112 (3) (2015) 669–672.
