# Supplementary material for: Network percolation reveals adaptive bridges of the mobility network response to COVID-19
Source: PLoS One. 2021 Nov 9;16(11):e0258868. doi: 10.1371/journal.pone.0258868 (PMC8577732; doi:10.1371/journal.pone.0258868)
Supplement: Attachment — Submitted filename: Response to Reviewers.pdf [file pone.0258868.s002.pdf]

Comment in Dark Grey
Response in Blue
Action in Green

Journal Requirements:
When submitting your revision, we need you to address these additional requirements.

1. Please ensure that your manuscript meets PLOS ONE's style requirements, including those for file naming. The PLOS ONE style templates can be found at

https://journals.plos.org/plosone/s/file?id=wjVg/PLOSOne_formatting_sample_main_body.pdf and

https://journals.plos.org/plosone/s/file?id=ba62/PLOSOne_formatting_sample_title_authors_affiliations.pdf

We have changed the format of our manuscript to meet PLOS ONE's style requirements.

2. We note that you have indicated that data from this study are available upon request. PLOS only allows data to be available upon request if there are legal or ethical restrictions on sharing data publicly. For more information on unacceptable data access restrictions, please see http://journals.plos.org/plosone/s/data-availability#loc-unacceptable-data-access-restrictions.

In your revised cover letter, please address the following prompts:

a) If there are ethical or legal restrictions on sharing a de-identified data set, please explain them in detail (e.g., data contain potentially sensitive information, data are owned by a third-party organization, etc.) and who has imposed them (e.g., an ethics committee). Please also provide contact information for a data access committee, ethics committee, or other institutional body to which data requests may be sent.

b) If there are no restrictions, please upload the minimal anonymized data set necessary to replicate your study findings as either Supporting Information files or to a stable, public repository and provide us with the relevant URLs, DOIs, or accession numbers. For a list of acceptable repositories, please see http://journals.plos.org/plosone/s/data-availability#loc-recommended-repositories.

We will update your Data Availability statement on your behalf to reflect the information you provide.

Cuebiq, a leader of location intelligence, provides de-identified and privacy-enhanced mobility data for academic research and humanitarian efforts only. All researchers using Cuebiq data were bound by contract not to share the data with anybody else or to try to de-identify it. Additional ethical oversight: we obtained IRB exemption to use the mobility data from the Northeastern IRB office through protocols # 21-02-09.

3. We note that Figures 1-5, S4, S6 in your submission contain map images which may be copyrighted. All PLOS content is published under the Creative Commons Attribution License (CC BY 4.0), which means that the manuscript, images, and Supporting Information files will be freely available online, and any third party is permitted to access, download, copy, distribute, and use these materials in any way, even commercially, with proper attribution. For these reasons, we cannot publish previously copyrighted maps or satellite images created using proprietary data, such as Google software (Google Maps, Street View, and Earth). For more information, see our copyright guidelines: http://journals.plos.org/plosone/s/licenses-and-copyright.

We require you to either (1) present written permission from the copyright holder to publish these figures specifically under the CC BY 4.0 license, or (2) remove the figures from your submission:

3.1. You may seek permission from the original copyright holder of Figure 1-5, S4, S6 to publish the content specifically under the CC BY 4.0 license.

We recommend that you contact the original copyright holder with the Content Permission Form (http://journals.plos.org/plosone/s/file?id=7c09/content-permission-form.pdf) and the following text:

"I request permission for the open-access journal PLOS ONE to publish XXX under the Creative Commons Attribution License (CCAL) CC BY 4.0 (http://creativecommons.org/licenses/by/4.0/). Please be aware that this license allows unrestricted use and distribution, even commercially, by third parties. Please reply and provide explicit written permission to publish XXX under a CC BY license and complete the attached form."

Please upload the completed Content Permission Form or other proof of granted permissions as an "Other" file with your submission.

In the figure caption of the copyrighted figure, please include the following text: “Reprinted from [ref] under a CC BY license, with permission from [name of publisher], original copyright [original copyright year].”

3.2. If you are unable to obtain permission from the original copyright holder to publish these figures under the CC BY 4.0 license or if the copyright holder’s requirements are incompatible with the CC BY 4.0 license, please either i) remove the figure or ii) supply a replacement figure that complies with the CC BY 4.0 license. Please check copyright information on all replacement figures and update the figure caption with source information. If applicable, please specify in the figure caption text when a figure is similar but not identical to the original image and is therefore for illustrative purposes only.

The following resources for replacing copyrighted map figures may be helpful:

USGS National Map Viewer (public domain): http://viewer.nationalmap.gov/viewer/

The Gateway to Astronaut Photography of Earth (public domain): http://eol.jsc.nasa.gov/sseop/clickmap/

Maps at the CIA (public domain): https://www.cia.gov/library/publications/the-world-factbook/index.html and https://www.cia.gov/library/publications/cia-maps-publications/index.html

NASA Earth Observatory (public domain): http://earthobservatory.nasa.gov/

Landsat: http://landsat.visibleearth.nasa.gov/

USGS EROS (Earth Resources Observatory and Science (EROS) Center) (public domain): http://eros.usgs.gov/#

Natural Earth (public domain): http://www.naturalearthdata.com/

We used the county division boundary shapefile from the link below:

https://www.arcgis.com/home/item.html?id=ff53e8fb54554031ace44ce9c1b119af.

The shapefile is produced by the U.S. Census Bureau and it states that: this work is licensed under a Creative Commons by Attribution (CC BY 4.0).

4. Please include captions for your Supporting Information files at the end of your manuscript, and update any in-text citations to match accordingly. Please see our Supporting Information guidelines for more information: http://journals.plos.org/plosone/s/supporting-information.

We have now included captions for the Supporting Information and update the in-text citation for the SI from the main manuscript.

Please use the space provided to explain your answers to the questions above. You may also include additional comments for the author, including concerns about dual publication, research ethics, or publication ethics. (Please upload your review as an attachment if it exceeds 20,000 characters)

Reviewer #1: This paper uses mobility traces collected from volunteers to detect differences in human mobility patterns between time periods immediately preceding and after the introduction of stay-at-home-orders due to the COVID-19 pandemic. The analysis performed by the authors focuses on graphs constructed by the amount of inter-county travel in the United States and the partitioning thereof. The authors find that the introduction of stay-at-home orders caused short term disruptions in the mobility network and that critical links to graph partitioning could be determined.

Generally, the topic is resonates with the times. It is clearly an important topic and will be of general interest to readers. The findings, while not altogether surprising provide an interesting framework for discussion of the role of human mobility in the spread of disease and how network analysis can be used to track diseases and possibly influence policy to better control the spread.

The idea of using the framework of phase transitions and finding q_c is an interesting one and the numerical analysis provided is solid. Similarly, the randomization steps taken to ensure that these are a product of human behavior and not merely products of a random network lend credibility to the work.

We appreciate Reviewer #1's summary of our work and these valuable suggestions. As the reviewer pointed out, we believe the percolation framework originated from network science is highly applicable to the inter-county human mobility network. Furthermore, there is a dearth of previous studies applying such methodology on the dynamic network with consideration of mobility fluctuations. By demonstrating the network percolation patterns, this study could be a stepping-stone for more complicated modeling and useful for policymakers. We've revised our manuscript and addressed the issues raised by Reviewer #1. We hope that our responses and the revised manuscript will meet the high standard of the journal.

However, there are things in the paper that are poorly explained that could stand better explanation.

1. It is not clear what the "median location" of a person is or how frequently these locations are computed. The paper rests on this idea, and yet it is not fully explained. If these are continuous GPS traces, is it the location at which the person is at noon? Are the GPS locations taken every 15 minutes and this is in fact the "average" location?

We thank the reviewer for this comment. The main motivation of the 'median location' is that it helps us determine which local geographic division (in our case, the county) that any user is primarily located within every 24 hours. The raw GPS data of each user has a varying sampling rate. Since most data points are generated when Apps in smart devices are launched or in use, the temporal granularity is usually in each second. The high temporal granularity results in a large quantity of data (over thousands of points) for some users. There are a few sophisticated algorithms previously developed such as the TimeGeo[1] framework or duration weighted averaging as a trajectory estimation method[2]. These methods are very effective when the number of users is small, but can be overly computationally expensive for tens of millions of users each day. Since the spatial resolution (i.e., on the county level) is relatively low and the geographical scale covers the entire U.S. in our analysis, we used the most straightforward and efficient method to compute the primary locations daily, i.e., computing the medians of longitude and latitude.

2. Similarly, percolation and phase transition theory come from physics, which is not the core audience of this paper, a brief introduction to what this is and what to look out for/why it is appropriate would help the reader immensely.

We appreciate this comment and agree with the reviewer. We have added a brief introduction on the background of percolation theory and its relevance to the study of human mobility and COVID-19 to address such issue. The text we have added can be found on page 2:

In the realm of network research, percolation theories are critical for identifying hierarchical structures and determining any discontinuous phase transition inside complex systems. There have been various studies using the percolation process in modeling real-world phenomena

[1] Jiang, S., Yang, Y., Gupta, S., Veneziano, D., Athavale, S. and González, M.C., 2016. The TimeGeo modeling framework for urban mobility without travel surveys. *Proceedings of the National Academy of Sciences*, *113*(37), pp.E5370-E5378.
[2] Mousavi, S.M., Harwood, A., Karunasekera, S. and Maghrebi, M., 2017. Geometry of interest (GOI): spatio-temporal destination extraction and partitioning in GPS trajectory data. *Journal of Ambient Intelligence and Humanized Computing*, *8*(3), pp.419-434.

such as traffic and routing networks[3,4] , the spread of infectious disease[5] [6], and cascading dynamics[7,8] .The bond percolation process has been commonly used in the epidemic modeling field entails deleting links at various cut-off values in order to find the critical threshold at which the network undergoes a structural shift such as break down of the large network into smaller components[9] . Since the presence of a link implies infection, such a threshold is closely tied to the emergence and disappearance of giant disease clusters. In the context of epidemic modeling, utilization of percolation theory combined with real-world mobility data can facilitate understanding the hierarchy of dynamic mobility networks and thus unearth the structure transition patterns [10].

.

3. Eyeballing Fig.2 and Fig 4. it doesn't look like the timing of the upswing is particularly different but the authors make the assertion on page 4/10 that these are significantly different. A stronger statement in that direction or justification would be nice.

We appreciate this comment and just as the reviewer pointed out, the upswing trends are quite similar for the time series plot in Fig. 2(g) and Fig.4 (e). However, in Fig (2)g, the mobility started the recovery after the curve reached its lowest point at the end of March 2020, the mobility began the recovery process immediately starting from the end of March 2020. However, for the percolation features in Fig.4 (a-b) and (d-e), there is a 3-week delay compared to Fig (2). The critical threshold stayed around the lowest point until the end of April before the first jump. Additionally, the recovery processes also differ in that the mobility metric recovery is a rather steady increase while the percolation features show less continuous with local spikes and drops. We agree with Reviewer 1 that clarification on the difference in such patterns would be needed and have added the following text on Page 7:

Noticeably, the recovery delays are different from the basic mobility metrics measured in Fig. 2 (g,h). Both mobility metrics immediately started to recover continuously after hitting the

---

[3] Li, D., Fu, B., Wang, Y., Lu, G., Berezin, Y., Stanley, H.E. and Havlin, S., 2015. Percolation transition in dynamical traffic network with evolving critical bottlenecks. *Proceedings of the National Academy of Sciences*, *112*(3), pp.669-672.
[4] Zeng, G., Li, D., Guo, S., Gao, L., Gao, Z., Stanley, H.E. and Havlin, S., 2019. Switch between critical percolation modes in city traffic dynamics. *Proceedings of the National Academy of Sciences*, *116*(1), pp.23-28.
[5] Wang, W., Tang, M., Stanley, H.E. and Braunstein, L.A., 2017. Unification of theoretical approaches for epidemic spreading on complex networks. *Reports on Progress in Physics*, *80*(3), p.036603.
[6] Barthélemy, M., Barrat, A., Pastor-Satorras, R. and Vespignani, A., 2005. Dynamical patterns of epidemic outbreaks in complex heterogeneous networks. *Journal of theoretical biology*, *235*(2), pp.275-288.
[7] Gleeson, J.P. and Cahalane, D.J., 2007. Seed size strongly affects cascades on random networks. *Physical Review E*, *75*(5), p.056103.
[8] Watts, D.J., 2011. A simple model of global cascades on random networks. In *The Structure and Dynamics of Networks* (pp. 497-502). Princeton University Press.
[9] Pastor-Satorras, R., Castellano, C., Van Mieghem, P. and Vespignani, A., 2015. Epidemic processes in complex networks. *Reviews of modern physics*, *87*(3), p.925.
[10] Li, M., Liu, R.R., Lü, L., Hu, M.B., Xu, S. and Zhang, Y.C., 2021. Percolation on complex networks: Theory and application. *Physics Reports*.

bottoms at the end of March. In contrast, the percolation features suggest that the overall network stayed at the low point for three more weeks before first experiencing a jump. The discrepancy again suggests that the recovery of the percolation criticality of human mobility follows a phase transition process rather than continuous change.

4. Similarly, the line in Fig. 4 for "mean value - after" is clearly only the mean value for some time-window after, but it is not clear what that window is.

We thank the reviewer for this comment. The 'before' or 'after' in Fig.4 refers to the time window before or after the national emergency declaration that was issued on March 13th, 2020. This was also shown in Fig. 1 with the same vertical line. To further eliminate any confusions, we have updated the caption of Fig. 4 to the following text:

Fig. 4 The changes of the percolation metrics using a 7-day moving window to smooth out the weekend/weekday fluctuations. **a** and **b** the changes of q_c with time and the median edge weights of the largest component which reflects the connection strength. **c**, the changes in the sizes of the largest strongly connected component for undirected graphs. **d, e** and **f** show the directed graph's scenario for q_c, median edge weight and largest component node size. Grey lines indicate the time series of the undirected graphs, while blue ones are for directed graphs. The red vertical line highlights the time of the national emergency declaration (March 13 ,2020) while the blue and red horizontal lines indicate the mean value of each feature before and after the declaration.

5. The significance of the "critical links" discussed on page 5 is somewhat dubious to me. It seems that the weighting on a link does not change drastically from time period to time period, and thus it's location in an ordered list of links weights would not shift drastically. Thus, a link that causes the "important" partition once is relatively likely to be in the same place again. I am not sure how the identification of this fact helps, since the remove of links in the network is a numerical not factual exercise.

We appreciate this comment and would like to elaborate more on the determination of critical links. Firstly, the link weight (number of people traveling between two counties) does change from time to time. This is shown In Fig. 2 (g) (also attached below), and the grey shaded area represents the interquartile range which indicates the variability across different links. When it comes to the mobility reaction of COVID-19, studies already showed discrepancies among different types of neighborhoods[11]. This could also be seen below that around mid-May, certain links already fully recovered while some others are still about a 20% below the level before the pandemic. Since different communities had different infection rates at different times and also responded to the stay-at-home order differently, the likelihood of having a fixed set of few critical links would be rather low. This point is also discussed in the SI. Secondly, we agree with the reviewer that such a determination process is not a factual exercise. Our work follows prior

[11] Coven, J. and Gupta, A., 2020. Disparities in mobility responses to COVID-19. *New York University*.

theoretical studies and in hope of finding important bridges. For instance, a previous study[12] used this method in finding the link that causes the traffic jam in an hourly updated transportation network and marks the efficacy of such an approach. Our objective in determining the critical links is to highlight the network structure shifts at different times.

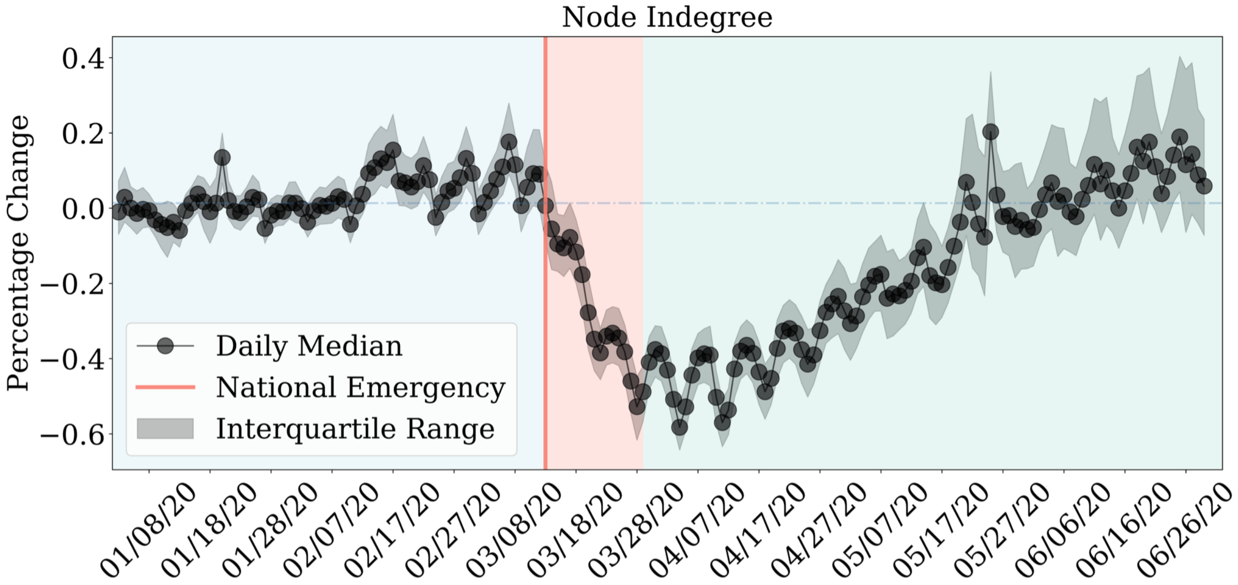

6. County size might vary dramatically across the united states. While restricting a user to one county per day might work in some places, I would assume that there are places in the united states for which users travel in multiple counties in a given day regularly. The "one county per day" restriction seems unnecessarily limiting. How would things have changed if the time granularity were an hour? I suppose this is related to Page 2: "We average the networks from 7 days" but it is not clear what that means either, how does that averaging happen?

We appreciate this comment, and we agree with the reviewer that geotagging all the GPS points to one county per day could be a limited method. The main objective of our study is to estimate the mobility between each county based on aggregated mobility data on each day. We made the comprise on spatial and temporal granularity so we are able to study the mobility network in the entire country. Also, we analyzed daily mobility patterns since the COVID-19 infection data is updated daily with uncertainties caused by the incubation period. A detailed explanation of averaging is included in Section Daily Fluctuation of Percolation Criticality of SI. We also acknowledge the limitations in our revised manuscript as following.

There are still some limitations in this study fostering some directions for further studies. First, the aggregated GPS data may not include individuals without smartphones and could overestimate those who have multiple devices. Moreover, our study focuses on the inter-county

[12] Li, D., Fu, B., Wang, Y., Lu, G., Berezin, Y., Stanley, H.E. and Havlin, S., 2015. Percolation transition in dynamical traffic network with evolving critical bottlenecks. *Proceedings of the National Academy of Sciences*, *112*(3), pp.669-672.

mobility network only. The relation between local mobility dynamics and the spread of COVID-19 also merits scrutiny and can be analyzed by more comprehensive and detailed modeling of people's movements locally. Such as map-matching trajectory to POIs with modeling of temporal visitation patterns. Finally, the aggregation and compression of mobility data presented in this study limits to only one county. An opportunity for future work is to formulate methods that can capture the detailed spatiotemporal movements of each user.

Smaller issues that need addressing:

1. On page four, in the middle: "Despite the network metrics steadily recovered" has grammatical issues, as does the rest of that paragraph.

We thank the reviewer for this comment and have revised the paragraph to the following:

The phase transition offers a unique and crucial perspective on how to manage people's movement during a pandemic. Despite the fact that mobility changes on a continual basis, the complex networks produced by human mobility have been found to undergo sudden phase transitions. This suggests that a subtle shift in mobility could lead to wide-scale reconnections of human mobility networks, resulting in the spread and resurgence of COVID-19 across broad geographies.

2. Final paragraph of page 4/10 has at missing \ in the LaTeX (bad section heading)

We thank the reviewer for pointing this out, and we have fixed this issue.

3. The first and last paragraphs of page 6/10 seem to be the same paragraph

We thank the reviewer for pointing this out, and we have fixed this issue.

4. In the abstract, I don't understand "The mobility network becomes vulnerable and prone to reach its criticality"

We thank the reviewer for this comment. The sentence was written to highlight our finding that the importance of critical threshold. A smaller value of such a threshold means with fewer travellers being restricted (links are removed), the network could become fragmented. We have revised this sentence to:

We investigate the bond percolation process by removing the weakly connected edges. As we increase the threshold, the mobility network nodes become less interconnected and thus experience surprisingly abrupt phase transitions.

Reviewer #2: The authors analyze the large-scale human mobility networks covering the first 6 months during the pandemic of COVID-19. They show, regardless of inherent complexity of human mobility, that these networks clearly exhibit abrupt phase transitions, in contrast with the

understanding of traditional percolation models where a continuous phase transition is expected. They develop new approaches to identify a set of recurrent critical links, connecting the giant component and the second-largest component, and show that these links are associated with network characteristics. This work shows a clear practical relevance and a general applicability in analyzing human mobility behaviors under interruptions of different kinds of infectious diseases. In addition, the paper is clearly written and easy to follow. It is a very good paper. Before recommending for publication, few minor comments are listed as follows.

We thank Reviewer #2 for the summary of our work and all the valuable suggestions. We appreciate Reviewer #2's recognition of our percolation-based approach in understanding the association between human mobility behaviors and transmission of COVID-19. We've revised our manuscript and clarified all the concerns raised by Reviewer #2. We hope that this response, as well as the revised version of our submission, will address the concerns mentioned by Reviewer #2 whilst also meeting the journal's high standards.

1 In the human mobility data, besides the two critical weight q_c and q_c2, do you observe more critical weights? This would for sure depend on a couple of factors like the total time span of the available data, the jump size and time window. I am wondering, within the available data, are there other critical weights in different parameter settings?

We appreciate this comment and there is indeed a great deal of research studies that have been devoted to the definition and selection of the percolation threshold. Since previous studies[13] already highlighted that human travel flow heavily affects the transmission rate on a global scale. Hence our critical weights are modelled and selected using the network edge weight which is the mobility flux between any two nodes (counties). As the reviewer suggested, there are other potential critical weights to be studied, especially on other dimensions. For instance, we could model the flux reduction rate or recovery rate between each pair of counties. We hope that would be a sequel to this study and in fact we are currently working on another study exploring the critical weights on different scales.

2 The results shown in Fig 2 indeed align with intuitive understanding that after the release of stay-at-home-orders, human mobility would decline. I am wondering are there variability and dependence with other factors like selected time window, either right after the declaration or even later than the studied one. In addition, are there some relevance with the daily infections? For instance, an increased daily infection would reinforce the emergency of current situation and mobility might accordingly drops.

[13] Kraemer, M.U., Yang, C.H., Gutierrez, B., Wu, C.H., Klein, B., Pigott, D.M., Du Plessis, L., Faria, N.R., Li, R., Hanage, W.P. and Brownstein, J.S., 2020. The effect of human mobility and control measures on the COVID-19 epidemic in China. *Science*, *368*(6490), pp.493-497.

We thank the reviewer for this comment. In fact, we had the exact same thoughts as Reviewer #2 when obtaining the results, and several studies[14][15] already validated the strong correlation between control measures and reduction of infection. However, in our sequel study to the current one, we plan to use external data such as SafeGraph which contains detailed social distancing metrics to furthermore model the relationship between human mobility behavior and the spread of COVID-19.

3 At the current presentation, the resolution of Fig 1 is lower than other figures. It would be easier to follow with a higher resolution.

We appreciate this comment and as the reviewer suggested we have updated the resolution of Fig. 1 in the latest version.

4 At page 3, Fig3b and Fig3c mismatch the text description. Last paragraph in page 4 is intended to be highlighted? At page 5, network structure instead of stricture. At page 2, please check Fig. 2b one.

We thank the Reviewer #2 for these comments, and we have thoroughly examined the issues raised by Reviewer #2 fixed all the typos.

[14] Xiong, C., Hu, S., Yang, M., Luo, W. and Zhang, L., 2020. Mobile device data reveal the dynamics in a positive relationship between human mobility and COVID-19 infections. *Proceedings of the National Academy of Sciences*, *117*(44), pp.27087-27089.

[15] Yabe, T., Tsubouchi, K., Fujiwara, N., Wada, T., Sekimoto, Y. and Ukkusuri, S.V., 2020. Non-compulsory measures sufficiently reduced human mobility in Tokyo during the COVID-19 epidemic. *Scientific reports*, *10*(1), pp.1-9.
