# Supplementary material for: Network percolation reveals adaptive bridges of the mobility network response to COVID-19
Source: PLoS One. 2021 Nov 9;16(11):e0258868. doi: 10.1371/journal.pone.0258868 (PMC8577732; doi:10.1371/journal.pone.0258868)
Supplement: Attachment — Submitted filename: Response to Reviewers.pdf [file pone.0258868.s003.pdf]

Comment in Dark Grey
Response in Blue
Action in Green

Reviewer #1: This paper continues to be an interesting paper and I look forward to seeing it published. The authors have addressed all my previous concerns but one that I would like to see addressed. In the authors response to my previous review, they explained that they calculated "median location" as the median of the latitude and longitude. It is easy to see that this may result in a location the user has not visited at all or spends very little time in (my own median location is likely somewhere on a highway in a county between my home county and work county). Nevertheless, I understand that the method is borne of necessity given the scale of the dataset and do not take over much issue with it. What I would like to see is some text in the published version of the paper that explains that this is how they have calculated location for a user. The current discussion is a single sentence that reads "We find the median of the locations from each user's daily movement trajectory and compute the primarily located county on each day(see Fig 1a)." which I feel is insufficient to explain what they have done. The paragraph they included in the response to reviewers is great and compelling, but I would be satisfied with a single parenthetical: "(i.e., the location of the medians of longitude and latitude)." It is a truly minor change. However, as the whole paper rests on where a user is considered to be, a strong understanding of how it is derived is crucial.

We thank the reviewer for this comment and regret that we had missed the reviewer's suggestion before. We agree with the reviewer; it is a crucial piece of information. We have added the text suggested by the reviewer.

Reviewer #2: The authors' response and modifications in the MS have clarified nearly all the concerns. A remaining issue is that after the replacement of fig1, it is still difficult to clearly read info in fig1, for example user labels in fig1b. After this issue has been addressed, I opt for a recommendation for publication.

We thank the reviewer for this comment and regret that Figure 1 was not as clear as it could be. We have updated the figure and particularly made the labels for users more readable. I hope the reviewer will find the figure more accessible.
